# Supplementary material for: Intra-serotype variation of Streptococcus pneumoniae capsule and its quantification
Source: Microbiol Spectr. 2025 Feb 14;13(4):e03087-24. doi: 10.1128/spectrum.03087-24 (PMC11960111; doi:10.1128/spectrum.03087-24)
Supplement: Supplemental material 1 — Detailed protocol for CPS immunoblot. [file spectrum.03087-24-s0001.pdf]

Capsular Immunoblot Protocol

Table of Contents

Materials and Apparatus………………………………………………………………………………… 2

Section I: Preparation for the Immunoblot……………………………………………………………………………… 3

I. Optimization - Primary Antibody Precipitation
II. Optimization - Triplicate Sample for Each Strain
III. Optimization - Reference Strain
IV. Strain Sample Preparation – Protein Concentration & Immunoblot Samples
V. Optimization - Equalized Loading by Protein Concentration
VI. Purified CPS Ladder
VII. Optimization - 1:2 Purified CPS Ladder

Section III: Immunoblot Application, Washing, Imaging, and Analysis…………………………………………………………………………………6

I. Immunoblot Device Assembly
II. Applying Samples to the Immunoblot
III. Applying the Primary & Secondary Antibodies to the Immunoblot
IV. Developing & Imagining the Immunoblot
V. Densitometry Analysis

Materials & Apparatus

Reagents & Antibodies

- 1X PBS
- 1% Triton X-100 in 1X PBS
- 0.1% Tween in 1X PBS (PBST)
- 3.33 μg/μL Proteinase K in 1X PBS (Denville Scientific, Cat.No. CB3210-5)
- Proteinase inhibitor: 1 tablet cOmplete™, EDTA-free Protease Inhibitor Cocktail in 1.5 mL 1X PBS (Roche, Ref# 11836170001)
- Thermo Scientific Pierce BCA Protein Assay Kit
- Merck or Pfizer Serotype Specific Purified Polysaccharide, acquired through American Type Culture Collection (ATCC).
  - Serotype 3 (Merck): ATCC # 17-X
  - Serotype 4 (Merck): ATCC # 173-X
  - Serotype 6A (Pfizer): ATCC #14-X
  - Serotype 23F (Pfizer): ATCC # 103-X
- 3% Milk in 0.1% PBST
- Statens Serum Institute Diagnostica Serotype-Specific Typing Serum in 0.1% PBST
  - anti-Serotype 3 (SSI Ref# 16746, rabbit, 1:40,000 in 0.1% PBST)
  - anti-Serotype 4 (SSI Ref# 16747, rabbit, 1:40,000 in 0.1% PBST)
  - anti-Serotype 6A (SSI Ref# 16900, rabbit, 1:40,000 in 0.1% PBST)
  - anti-Serotype 23F (SSI Ref# 16913, rabbit, 1:40,000 in 0.1% PBST)
- Anti-Rabbit IgG-Conjugated Horseradish Peroxidase (Invitrogen, Ref# G21234, goat, 1:5000 in 0.1% PBST)
- Thermo Scientific Super Signal West Femto Maximum Sensitivity Substrate

Other Materials

- GE Healthcare Life Sciences Whatman Qualitative Filter Paper Grade 3
- GE Healthcare Life Sciences Amersham 0.45 μm Nitrocellulose Membrane

Apparatus

- Heating Block/Incubator
- Blot Box
- Cole Palmer Minifold II Slot-Blot Manifold
- Plate Shaker
- iBright Imaging System
- Vacuum Filtration System

Section I: Preparation for the Immunoblot

I: Optimization - Primary Antibody Precipitation

Pneumococcal typing serum can be polyclonal and bind to non-capsular proteins. In such cases, they must be pre-treated with an acapsular strain to saturate out any non-capsule specific antibodies. While an acapsular strain of a certain serotype may not be readily available, an acapsular strain from a different serotype can still successfully precipitate out some, if not all, non-capsule specific antibodies. When running immunoblots, it is imperative to also visualize the acapsular strain used in precipitating out non-specific antibodies in the typing serum, such that signal intensities due to antibody binding to non-capsule proteins can be subtracted out from the experimental strains, yielding the true data.

1. Grow an unencapsulated pneumococcal strain of the desired serotype to mid-log phase at 37°C.
2. Centrifuge 1-2 mL of the unencapsulated bacterial culture at 13,500 g for 1 minute. Remove the supernatant.
3. Resuspend the pellet in 1 mL of 1X PBS, and centrifuge again. Remove the supernatant.
4. Resuspend the pellet again in 1 mL of 1X PB, and add the desired serotyping antiserum to create a 1:200 dilution of the typing serum.
5. Incubate the mixture at 37°C for 1 hour, inverting the mixture occasionally.
6. After 1 hour, centrifuge the mixture at 13,500 xg for 1 minute.
7. Carefully transfer the supernatant to a new Eppendorf, taking as little of the pellet as possible. Please note, the supernatant IS the typing serum.
8. Centrifuge again at 13,500 xg for 1 minute, and transfer 800 µL of the solution to a new Eppendorf. Less of the solution is taken to avoid taking up any remaining bacteria.
9. Centrifuge again at 13,500 xg for 1 minute, and transfer 600 µL of the solution to a new Eppendorf. This is the final mixture of the typing serum that will act as the primary antibody for the immunoblotting assay.
10. Dilute the typing serum again at 1:200 to yield a final concentration of 1:40000 for the primary antibody.
11. Test out the primary antibody with a sample of the experimental strain and a sample of the unencapsulated strain to observe the efficacy of absorbing out non-capsule specific antibodies with the unencapsulated strain. A weak or nonexistent signal should appear for the unencapsulated strain sample. If a strong signal appears, a repeat of incubation with the unencapsulated strain and previous steps may be necessary.

II: Optimization - Triplicate Sample for Each Strain

- For each strain of interest, it is recommended that three samples are made from independently-grown bacterial culture. This helps account for sample-to-sample fluctuations of CPS quantity due to growth conditions.
- Strains do not have to be grown on the same day.
- However, triplicate samples should be loaded on the same blot.

III: Optimization - Reference Strain

- To compare CPS quantity of strains across different blots, a reference strain with triplicate samples should be loaded onto each blot. By normalizing the CPS quantity of each strain to that of the reference during densitometry analysis, CPS quantity of strains across different blots and experiments can be compared to each other.
- Because the primary and secondary antibodies can be reused, intensities of signals may vary from blot to blot, as well as experiment to experiment.

IV: Strain Sample Preparation - Protein Concentration & Immunoblot Samples

1. Grow all bacterial strains to the desired optical density. For most serotypes, mid-log phase will suffice. For mucoid strains or those that do not pellet well, a late mid-log phase may work better.
2. Centrifuge 2 mL of each sample strain at 13,500 xg for 1 minute. Remove the supernatant.
    a. For mucoid strains, an additional step of resuspending the pellet in 1 mL of 1X PBS and pelleting is necessary. Remove the supernatant.
3. Lyse each sample with 400 µL of the lysing solution, 1% Triton X-100 in PBS. Leave at room temperature until the sample mixture turns from turbid to clear, indicating that lysis has occurred. Samples can be frozen at -20°C at this point for continuation on a different day if necessary.
4. Transfer 180 µL of the lysed mixture to an Eppendorf for the protein measurement sample and another 180 µL of the mixture to another Eppendorf for the immunoblot sample.
    a. In the protein measurement sample, add 30 µL of the proteinase inhibitor.
    b. In the immunoblot sample, add 30 µL of 3.33 µg/µL Proteinase K in 1X PBS.
5. Incubate the samples. After incubation, samples can be frozen again at -20°C if necessary.
    a. The protein measurement samples need to be incubated at 37°C for 5 minutes.
    b. The immunoblot samples need to be incubated at 65°C for 15 minutes.
6. Dilute the final immunoblot samples to 1:100 in PBST. To yield a representative sample, dilute at least 40 µL in 4 mL of PBST.
    a. For strains with too intense signals, try a 1:1000 dilution.
    b. For strains with too weak signals, try a 1:10 dilution.

V: Optimization - Equalized Loading by Protein Concentration

- Proceed with protein measurement using the Pierce BCA Protein Assay Kit as directed by the kit instructions.
- Equalize the loading volume of the samples based off of the protein measurement for each sample.
    - Equalizing samples to 50 ng of protein is a good starting point.
    - Loading may need to be adjusted for certain strains depending on their blot signal intensities, so not all strains on a blot may be equalized to 50 ng of protein.

<u>VI: Purified CPS Ladder</u>

1. Dilute a series of polysaccharide standard using the Merck or Pfizer serotype-specific purified polysaccharide in 1X PBS.
    a. An operating range between 512 μg/mL to 0.25 μg/mL is typically used.
2. Depending on the desired effect, a dilution series of 1:2 to 1:5 can be successful in capturing the range of the immunoblot samples.

<u>VII: Optimization - 1:2 Purified CPS Ladder</u>

- A 1:2 dilution series of the purified CPS standard yields the most consistent curve for quantification analysis during densitometry.
- Strain samples should be adjusted to fit into the sensitive, linear range of the purified CPS ladder.
    - In this linear range, quantification data tends to be replicable and consistent, allowing for comparisons across blots and experiments.

Section II: Immunoblot Application, Washing, Imaging, and Analysis

I: Immunoblot Device Assembly

1. Assemble the vacuum slot-blot device as instructed by device manual.
    a. For example, the Cole Palmer Minifold II Slot-Blot Manifold is assembled as follows:
        i. Bottom acrylic piece
        ii. Leather piece
        iii. Plastic barrier piece
        iv. Middle acrylic piece with run-off channels
        v. 2 x Whatman filter paper grade 3
        vi. 0.45 µm nitrocellulose membrane
        vii. Top acrylic piece with sample wells
2. On top of the middle acrylic piece, place two layers of the Whatman filter paper grade 3, cut to size, and dampen with 1X PBS.
3. Place the 0.45 µm nitrocellulose membrane on top of the dampened filter papers and align with the sample wells as best fit.
4. Place the top acrylic piece over the nitrocellulose membrane, and lock the device in place before connecting to a vacuum hose.

II: Applying Samples to the Immunoblot

1. Turn on the vacuum. Observe that the vacuum is running successfully and pulling liquid from the device.
2. Load the purified polysaccharide ladder and the strain samples to their designated wells.
    a. To make the process faster, pre-load a 96-well plate with the purified polysaccharide ladder and strain samples, then load onto the device.
3. Once the ladder and the samples have been suctioned through the device and onto the membrane, wash each well with 250 µL 1X PBS.
4. After the wells appear dry, turn off the vacuum.
5. Disconnect the vacuum hose, and carefully disassemble the device.
6. Transfer the membrane carefully to a blot box with the sample-side facing up.
7. Wash the slot-blot device with warm water for next use.

III: Applying the Primary & Secondary Antibodies to the Immunoblot

1. To the blot box with the nitrocellulose membrane, add enough 3% Milk in 0.1% PBST to allow the membrane to be suspended in the liquid, moving freely.
    a. This is to block off the membrane from reacting with the primary and secondary antibodies.
2. Place the blot box onto a plate shaker set to 60 RPM and incubate the membrane in the milk for 30 minutes at room temperature.
3. Remove and save the milk mixture, storing at 5°C. This can be re-used until mixture becomes discolored, chunky, or acrid.
4. Rinse the blot with 1X PBS with shaking for 5 minutes. Discard the PBS once done.

5. Add enough of the serotype-specific primary antibody to suspend the blot in the liquid, moving freely.
6. Incubate the membrane in the primary antibody for 30 minutes with shaking at room temperature.
7. Remove and save the primary antibody, storing at -20°C. This can be reused up to 5 times.
8. Rinse the blot with 0.1% PBST with shaking for 10 minutes. Discard the PBST. Repeat the rinse, and discard the PBST once more.
9. Add enough of the secondary antibody IgG-conjugated horseradish peroxidase to suspend the blot in the liquid, moving freely.
10. Incubate the blot in the secondary antibody for 30 minutes with shaking at room temperature.
11. Remove and save the secondary antibody, storing at 5°C. This can be reused up to 3 times.
12. Rinse the blot with 0.1% PBST with shaking for 10 minutes. Discard the PBST. Repeat the rinse, and discard the PBST once more.

IV: Developing & Imaging the Immunoblot

1. Follow the instructions for the ECL solution, and mix enough to yield 1 mL of signal amplifier per blot.
2. Apply the ECL solution to the blot by pipetting the solution over the blot, making sure to cover every part.
3. Using a tweezer, pick up the blot, allowing some of the ECL solution to drain off, and set onto the imaging platform.
    a. Make sure the blot is damp enough to stick to the platform, but not overly damp to have liquid pool over the camera and obstruct the imaging process.
4. Set the machine to acquire image for chemi blots, and run on auto-exposure.
    a. The auto-exposure should be around 1-3 seconds, and no more than 10 seconds.
    b. If the exposure time surpasses 10 seconds, there’s likely an error with the primary or the secondary antibody, where the antibody is too weak from repeat use.
        i. If this occurs, save the blot by resuspending in 0.1% PBST and refrigerate at 5°C.
        ii. Re-dilute new mixtures for the primary and secondary antibodies.
        iii. Go back to step 5 of **“Applying the Primary & Secondary Antibodies to the Immunoblot”** and repeat subsequent steps.
5. Acquire the image and save as a TIFF file, 600 dpi or highest resolution.

V: Densitometry Analysis

1. Perform densitometry analysis on the blots by using Image J or similar software.
    a. Please note that consistency in densitometry analysis is crucial to the data, i.e. marking the area under the curve consistently at the flat portion or curve.

2. Graph the area under the curve vs. purified CPS standard concentrations. The equation describing the curve of best fit will be the equation used to calculate the CPS of each sample.
    a. For a 1:2 dilution ladder, the linear portion of the curve of best fit is the most sensitive range of the ladder. Adjust the samples to this sensitive range.
    b. If a signal is present for the chosen unencapsulated strain used to saturate out unspecific antibody binding to non-capsular proteins, then the area under the curve value for the unencapsulated strain needs to be subtracted out from the value of the experimental strains.
3. Data can be presented in a number of ways, but the most intuitive would be to calculate the **amount of CPS per protein,** as the loading is based off of the protein concentration of each sample.
4. Normalize the CPS per protein value of each strain to the reference strain for comparison.
5. Normalize the CPS per protein value of the reference strain on each blot.
