# Supplementary material for: Intra-serotype variation of Streptococcus pneumoniae capsule and its quantification
Source: Microbiol Spectr. 2025 Feb 14;13(4):e03087-24. doi: 10.1128/spectrum.03087-24 (PMC11960111; doi:10.1128/spectrum.03087-24)
Supplement: Supplemental material 2 — Detailed protocol for dextran exclusion assay and Fiji macro. [file spectrum.03087-24-s0002.pdf]

Dextran-exclusion assay protocol

This protocol describes bacterial culture conditions and volumes for our experiments but can be adapted according to specific needs. The Dextran should be labelled with a fluorophore. FITC-Dextran is a widely available option.

Material

- Heating block or water bath, 37°C
- Heating block or water bath, 50-60°C
- Microscopy slides, washed with detergent
- #1.5 cover slip (0.16-0.19mm thickness)
- Clear nail polish
- Growth medium
- 1% agarose in growth medium (or in 1X PBS) 50-60°C

Fluorescent dyes

- Store at -20°C
- Dyes can be placed back into freezer and re-used
- 10mg/mL 2000kDa FITC-Dextran in PBS (Sigma-Aldrich, Ref# FD2000S)
- 1mM Nile red (ca. 0.3183mg/mL) in methanol (Sigma-Aldrich, Ref# 72485)

Solution to prepare for fluorescence microscopy

Staining Solution

- Growth medium (or 1X PBS)
- 2.5nM Nile red
    - 1mM Nile red can be pre-diluted 1:10 in growth medium (or 1X PBS)
- 2mg/mL 2000kDa FITC-dextran

Agarose-Dye-Mix for pad

- 1% agarose in growth medium or 1X PBS
- 2.5nM Nile red
    - 1mM Nile red can be pre-diluted 1:10 in growth medium (or 1X PBS)
- 2mg/mL 2000kDa FITC-dextran
- Keep at 50-60°C
    - Agarose pad should be prepared just when the bacteria are suspended in the Staining Solution. When left for too long, they dry out, which makes cover slip removal difficult

Preparation of microscopy samples

Bacterial liquid culture and harvest

- Grow bacteria to desired density, e.g. mid-log phase in broth
- Take 1mL culture and centrifuge at 8000xg, 1min
- Remove supernatant
- Re-suspend pellet in 100µL previously prepared *Staining Solution*

Agarose pad

- Pipette previously prepared *Agarose-Dye-Mix* on microscopy slide and immediately place cover slip
  - Agarose should spread and fill gap underneath cover slip completely and then immediately solidify
  - 75µL Agarose-Dye-Mix for 18x18mm coverslips
  - 100µL Agarose-Dye-Mix 22x22mm coverslips
- Slide off cover slip from agarose after it sets
  - Slides off easily within 2-5min of casting
- Air-dry pads for 30-60sec to dry excess moisture (fanning air speeds up the process)
  - The earlier the cover slip is removed the longer it takes for moisture to dry

Microscopy Slide preparation

- Add 5-10µL bacterial suspension in *Staining Solution* on new #1.5 cover slip
- Place bacteria bearing side on agarose pad and gently press on
- Seal coverslip edges with nail polish
  - Topcoat nail polish works well as it is very liquid and dries very fast

General tips

1. After prep, pictures of bacteria have successfully been taken for up to 3h
2. Work on room temperature. Woking on ice has two problems
   - Condensation on coverslip leads to moisture layer between glass and agarose, preventing immobilisation of bacteria
   - In quite some strains, placing bacteria on ice and then working on room temperature during microscopy seems to trigger lysis
3. Growth medium and 1X PBS work equally good in this protocol, and can be chosen dependent on experimental setup

The Fiji plugin

The Fiji plugin is a collection of macros and functions used to prepare the image and execute automated analysis for defined regions of interest (ROI). Below is a description of the most integral macros and functions. The plugin contains several other qualities of life functionalities which are annotated within the code but not described here.

1) After starting Fiji, load the plugin through `Plugins>Macros>Install…`
2) Prepare images for analysis by using `'Fix Channel Colors'` macro (can be initiated with `F5`) . Set channel colours, set image type, save image as TIF. After saving, the view is set to the red channel and greyscale colours. The line tool is selected.
3) Draw a line perpendicular to cell membrane surfaces, choose as many cells as needed. Pressing `t` will define the line as a ROI. The macro `'Rename and Save ROI Manager with Numbers'` (initiated with `F6`) will standardise the line length, and safe the coordinates of all ROIs in the same folder as the image file.
4) The macro `'Measure selected cells'` (initiated with `F7`) will measure capsule width and bacterial cell size and provide and output table which can be copied.

Global Variabels

```
var tab = " \t";
var sampledLineWidth = 5;   // how wide is the line for plotProfile
var sampledLineLength = 90; // how long is the line for plotProfile
```

These global variables set parameters for the entire macro. They define the width and length of the line used for `'plotProfile'` when measuring cell and capsule size. In this example, the line width is set to 5 pixels and the length to 90 pixels.

Fix Channel Colors

```
macro "Fix channel colors [F5]" {
    Property.set("CompositeProjection", "Sum");
    Stack.setDisplayMode("composite");
    Stack.setChannel(1);
    run("Cyan");
    Stack.setChannel(2);
    run("Grays");
    Stack.setChannel(3);
    run("Magenta");
    run("Save");
    roiManager("reset");
    roiManager("Show All");
    RoiManager.associateROIsWithSlices(false);
    RoiManager.restoreCentered(false);
    RoiManager.useNamesAsLabels(false);
    Property.set("CompositeProjection", "null");
    Stack.setDisplayMode("grayscale");
    Stack.setChannel(3);  // sets Nile red channel
    setTool("line");
```

This macro changes the display colour of various channels acquired during microscopy for better accessibility. The green channel (FITC-dextran) is set to cyan, the red channel (Nile red) to magenta, and the transmission light channel to grey. Display channels can be changed in the code as desired according to the range of channel colours offered by Fiji.
After setting the colours, it triggers a save-prompt for the altered picture, resets the ROI manager, and prepares the file for ROI selection by setting the display to grayscale and selecting the red channel. The line-drawing tool is then selected for defining ROIs.
It is important that the centre of the line falls within the central area of the bacterial cell. After drawing the line, the command “t” saves it as an ROI in the ROI manager.

Rename and Save ROI Manager with Numbers

```
macro "Rename and save ROI Manager with numbers [F6]" {
    for (roi = 0; roi < roiManager("count"); roi++) {
        roiManager("select", roi);
        newROIname = IJ.pad(roi + 1, 3);
        roiManager("rename", newROIname);
        remapLineFixedLength(sampledLineLength);  // OPTIONAL makes the line a standard
length
        roiManager("Update");  // OPTIONAL puts the updated ROI in the manager
    }

    if (getInfo("window.type") != "Image") exit("Make sure running macro from image
window.");
    dir = getDirectory("image");  // where to save results
    if (lengthOf(dir) < 1) exit("Make sure the image was opened from disk.");
    title = getTitleStripExtension();
    newName = dir + title + ".zip";
    roiManager("Save", newName);
}  // end "rename and save ROI Manager with numbers [F6]"

/* This function takes a straight line selection and redraws it at a fixed length with the
center position constant. Units are pixels. */

function remapLineFixedLength(newLength) {
    if (!(selectionType == 5)) exit("Requires straight line selection.");
    if (newLength <= 0) exit("Length must be positive.");
    Roi.getCoordinates(xpoints, ypoints);
    xc = (xpoints[0] + xpoints[1]) / 2;
    yc = (ypoints[0] + ypoints[1]) / 2;
    run("Measure");
    angle = newArray(2);
    angle[0] = getResult("Angle", nResults - 1) * PI / 180 + (PI / 2);
    angle[1] = angle[0] + PI;
    for (i = 0; i < 2; i++) {
        xpoints[i] = xc + newLength / 2 * sin(angle[i]);
        ypoints[i] = yc + newLength / 2 * cos(angle[i]);
    }
    makeLine(xpoints[0], ypoints[0], xpoints[1], ypoints[1]);
}  // end remapLineFixedLength
```

This macro renames the ROIs in the ROI manager with a numerical identifier for easier reference. It then adjusts the length of the line defining each ROI to a fixed pixel size as specified in the global variables. Finally, it saves all ROIs in the same directory as the image file. This prepares the image for automated measurement of cell size, dextran-exclusion zone, and capsule width calculation.

Measure Selected Cells

```
macro "Measure selected cells [F7]" {
    print("ROI#", tab, "redWidth", tab, "greenShadow", tab, "capsuleWidth", tab,
getTitle());
    for (roi = 0; roi < roiManager("count"); roi++) {
        roiManager("select", roi);
        remapLineFixedLength(sampledLineLength);
        measureEachChannel(roi, 0.5);  // Measure at intensity 0.5
    }
    selectWindow("Log");
}

function measureEachChannel(roiNum, widthAtIntensity) {
    errorFlag = "";
    maxWidthCell = 3;  // in um, the maximum width of a cell before an error flag is set
    remapLineFixedLength(sampledLineLength);
    setLineWidth(sampledLineWidth);  // reduce noise by widening the line
    sizeFactor = getSizeOfEachPointOnLine();  // how big is each point on the line
    getVoxelSize(pxwidth, pxheight, pxdepth, unit);  // only unit is used in the macro
    roiManager("Remove Channel Info");
    roiManager("Remove Slice Info");
    roiManager("Remove Frame Info");

    // Measure Nile red channel
    Stack.setChannel(3);  // Nile red channel is 3 in our images.
    profile = getProfile();
    normalizeArray(profile);
    redLeftPosition = findLeftValue(profile, widthAtIntensity);
    redRightPosition = findRightValue(profile, widthAtIntensity);
    redBacteriumWidth = abs(redRightPosition - redLeftPosition) * sizeFactor;
    if (redBacteriumWidth >= maxWidthCell) errorFlag = "red_too_wide ";

    // Measure FITC-dextran channel
    Stack.setChannel(1);  // FITC-dextran cannel is 1 in our images.
    profile = getProfile();
    normalizeArray(profile);  // it is an unusual approach to place the results in the
original array
    for (i = 0; i < profile.length; i++)  // inverts the values for channel 1
        profile[i] = 1 - profile[i];  // inverts the values for channel 1
    greenLeftPosition = findLeftValue(profile, widthAtIntensity);
    greenRightPosition = findRightValue(profile, widthAtIntensity);
    greenShadowWidth = abs(greenRightPosition - greenLeftPosition) * sizeFactor;

    capsuleWidth = (greenShadowWidth - redBacteriumWidth) / 2;

    print(roiNum + 1, tab, redBacteriumWidth, tab, greenShadowWidth, tab, capsuleWidth,
tab, errorFlag);

    run("Select None");
    selectWindow("Log");
}  // end measureEachChannel function

/*=============================================================================
Functions to identify positions that cross the desired threshold
- Expected input is an array normalized from 0 to 1 inclusive, but it will work on arrays
of any range as long as all elements are numbers.
- Returns "i" which is the position in the array of the first pixel intensity which crosses
the threshold.
- This needs to be converted to um using getVoxelSize and the length of the line. */

function findLeftValue(a, threshold) {
    for (i = 0; i < a.length; i++) {
        if (a[i] >= threshold) {
```

```
            leftIndex = i;
            i = 999999;  // exits loop; would be cleaner with repeat until, but this works
        }
    }  // for
    return (leftIndex);  // else return the position in the array of the edge
}  // end findLeftValue function

function findRightValue(a, threshold) {
    for (i = a.length - 1; i >= 0; i--) {  // get right
        if (a[i] >= threshold) {
            rightIndex = i;
            i = -1;  // exits loop
        }
    }
    return (rightIndex);  // else return the position in the array of the edge
}  // end findRightValue function
```

This macro measures the signal intensities in the green (FITC-dextran) and red (Nile red) channels for each stored ROI and translates these into a plot profile. The signal intensity is normalised from 0 to 1. To reduce background, the line defining the ROI is widened to five pixels and average intensities are calculated for each given point on the line. Subsequently, the macro analyses the plot profile from the left side of the x-axis to find the point at which the signal intensity reaches 0.5. It then repeats that step starting from the right side of the x-axis. Using the known pixel dimensions, it calculates the cell size (Nile red) and dextran-exclusion zone (FITC-dextran), and subsequently the capsule width using the formula:

$$capsule\ width = \frac{[dextran\ exclusion\ zone]\ - [cell\ size]}{2}$$

We chose to measure bacterial cell size and dextran-exclusion at a signal intensity of 0.5 to avoid highly fluctuating background signals.
The 'Stack.setChannel (X)' command is dependent on the specific order that channels are acquired by a microscope. If the red channel (Nile red) would be acquired in channel 3, this command must be changed to 'Stack.setChannel (3)'.
Values below 0.1µM should be considered to be 0, since the detection limit for green light is 200nm (therefore 100nm in our setup as we measure across the cell).

Multichannel Plot Profile graphical output

```
macro "Multichannel plot profile graphical output" {
    makeOutputTable = true; // Set to false if you don't want an output table
    processChannel = newArray(1, 0, 1, 0, 0, 0, 0, 0, 0, 0, 0, 0, 0, 0); // Choose which
channels to plot or not plot
    Stack.getDimensions(width, height, channels, slices, frames);
    original = getImageID();
    t = getTitleStripExtension();
    mergeString = "";
    newChannelNumber = 1;
    newExecution = true;

    for (c = 1; c <= channels; c++) {
        if (processChannel[c - 1]) {
            selectImage(original);
            Stack.setChannel(c); // Set channel
            Color.getLut(reds, greens, blues);
```

```
            if (makeOutputTable) {
                profile = getProfile();

                if (newExecution) { // Create a new output table
                    getPixelSize(unit, pixelWidth, pixelHeight);
                    run("Set Measurements...", "area redirect=None decimal=2");
                    run("Measure");
                    distances = newArray(profile.length);
                    pixelSize = getResult("Length", nResults - 1) / profile.length; // um
per pixel
                    for (d = 0; d < profile.length; d++)
                        distances[d] = d * pixelSize;
                    Table.create(t + "_results");
                    Table.setColumn(unit, distances);
                    newExecution = false;
                }

                // Normalize the profile values using custom functions
                maxProfileValue = getMaxValue(profile);
                minProfileValue = getMinValue(profile);
                for (i = 0; i < profile.length; i++) {
                    profile[i] = (profile[i] - minProfileValue) / (maxProfileValue -
minProfileValue);
                }

                // Invert the normalized profile for the green channel (assuming channel 1
is green)
                if (c == 1) {
                    for (i = 0; i < profile.length; i++) {
                        profile[i] = 1 - profile[i];
                    }
                }

                Table.setColumn("ch" + c, profile);
                Table.update();
            }

            // Plot profile of specific slice
            selectImage(original);
            Stack.setSlice(1); // Set the slice number you want to plot
            profile = getProfile();
            // Normalize and invert profile for plotting
            maxProfileValue = getMaxValue(profile);
            minProfileValue = getMinValue(profile);
            for (i = 0; i < profile.length; i++) {
                profile[i] = (profile[i] - minProfileValue) / (maxProfileValue -
minProfileValue);
            }
            if (c == 1) {
                for (i = 0; i < profile.length; i++) {
                    profile[i] = 1 - profile[i];
                }
            }
            Plot.create("Plot", "Distance", "Intensity", profile);
            Plot.show();
            run("Invert");
            Color.setLut(reds, greens, blues);
            rename("plot_ch" + c);
            mergeString = mergeString + "c" + newChannelNumber + "=" + getTitle() + " ";
            newChannelNumber++;
        } // process channel
    } // for each channel
    run("Merge Channels...", mergeString + " create");
    rename(t + "_plot");
    selectWindow(t + "_results");
}
```

```
// Function to get the maximum value in an array
function getMaxValue(array) {
    max = array[0];
    for (i = 1; i < array.length; i++) {
        if (array[i] > max) {
            max = array[i];
        }
    }
    return max;
} // end function to get maximum value

// Function to get the minimum value in an array
function getMinValue(array) {
    min = array[0];
    for (i = 1; i < array.length; i++) {
        if (array[i] < min) {
            min = array[i];
        }
    }
    return min;
} // end function to get minimum value
```

This macro visualises the plot profiles that are used to measure signal intensities in each channel. It processes selected channels, creates an output table with distances, and generates plot profiles for specific slices.
