# Supplementary figures and images for: Intra-serotype variation of Streptococcus pneumoniae capsule and its quantification
Source: Microbiol Spectr. 2025 Feb 14;13(4):e03087-24. doi: 10.1128/spectrum.03087-24 (PMC11960111; doi:10.1128/spectrum.03087-24)

# Supplementary Material 3

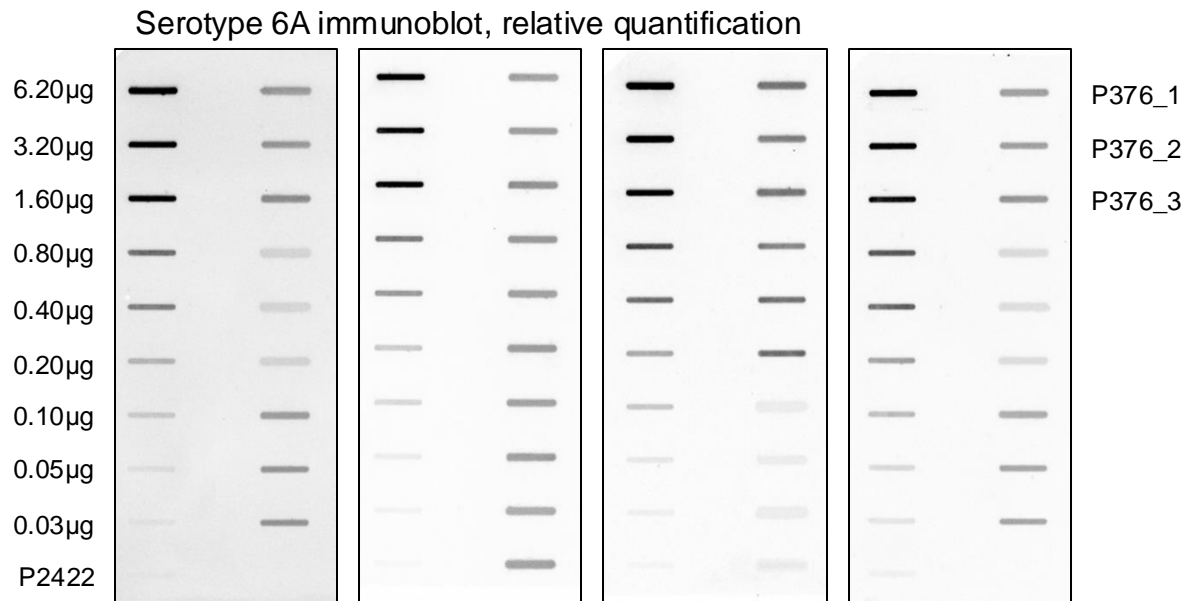

Supplementary Material 3 – Full blots of Fig S2A

Supplement: Supplemental material 3 — Full blots from Fig S2A. [file spectrum.03087-24-s0003.pdf]
