# Supplementary material for: Intra-serotype variation of Streptococcus pneumoniae capsule and its quantification
Source: Microbiol Spectr. 2025 Feb 14;13(4):e03087-24. doi: 10.1128/spectrum.03087-24 (PMC11960111; doi:10.1128/spectrum.03087-24)
Supplement: Supplemental figures — Fig. S1 to S9. [file spectrum.03087-24-s0004.pdf]

Fig S1

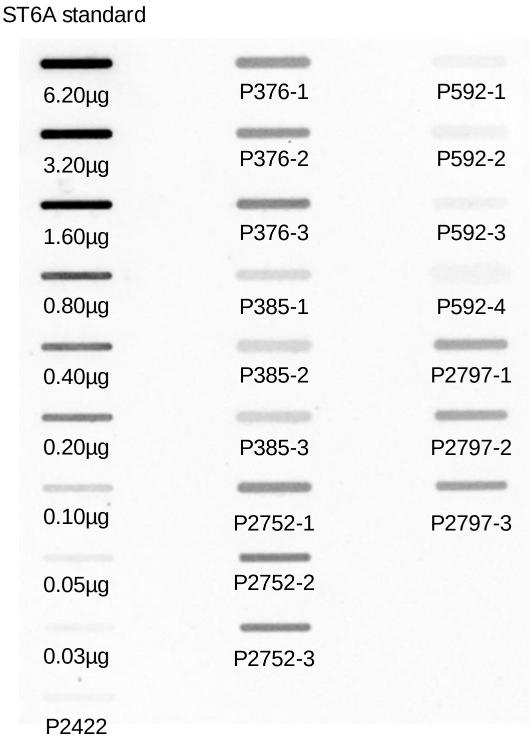

Supplementary Figure 1 Capsular polysaccharide immunoblot using a reference strain. Serotype specific purified polysaccharide standard (left lane, specific quantity indicated below signal) and samples (biological triplicates, middle and right lane) were transferred to a nitrocellulose membrane. Immunoblotting was performed using serotype-specific antibody. The standard was used to establish a sensitive range for densitometry, which is used to adjust sample loading accordingly. A reference strain (P376) was used to compare samples across blots with each other. P2422 is an unencapsulated control strain.

Fig S2

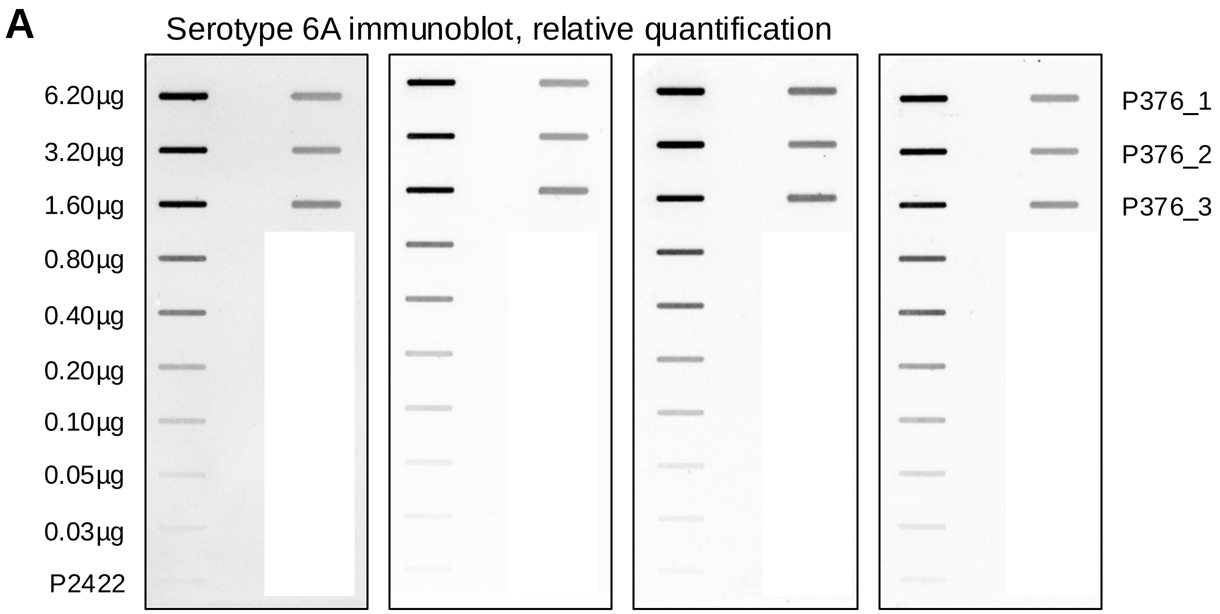

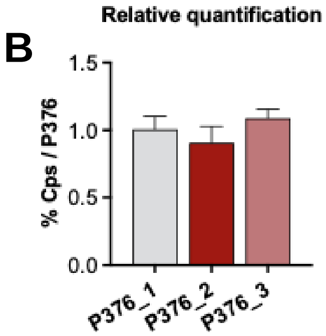

Supplementary Figure 2 Relative quantification in Capsular polysaccharide immunoblot. (A) The same sample from three independent experiments (right lanes) was analyzed by densitometry on four independent immunoblots. The standard (left lane) was used to assess whether samples are in sensitive range. Other samples on blots are covered by white boxes for clarity. Full blots can be found in Supplementary Material 3. (B) Densitometry results show low variability is observed in relative quantification approach. Standard deviation shown. Ordinary one-way ANOVA with multiple comparison performed. Non-significant differences.

Fig S3

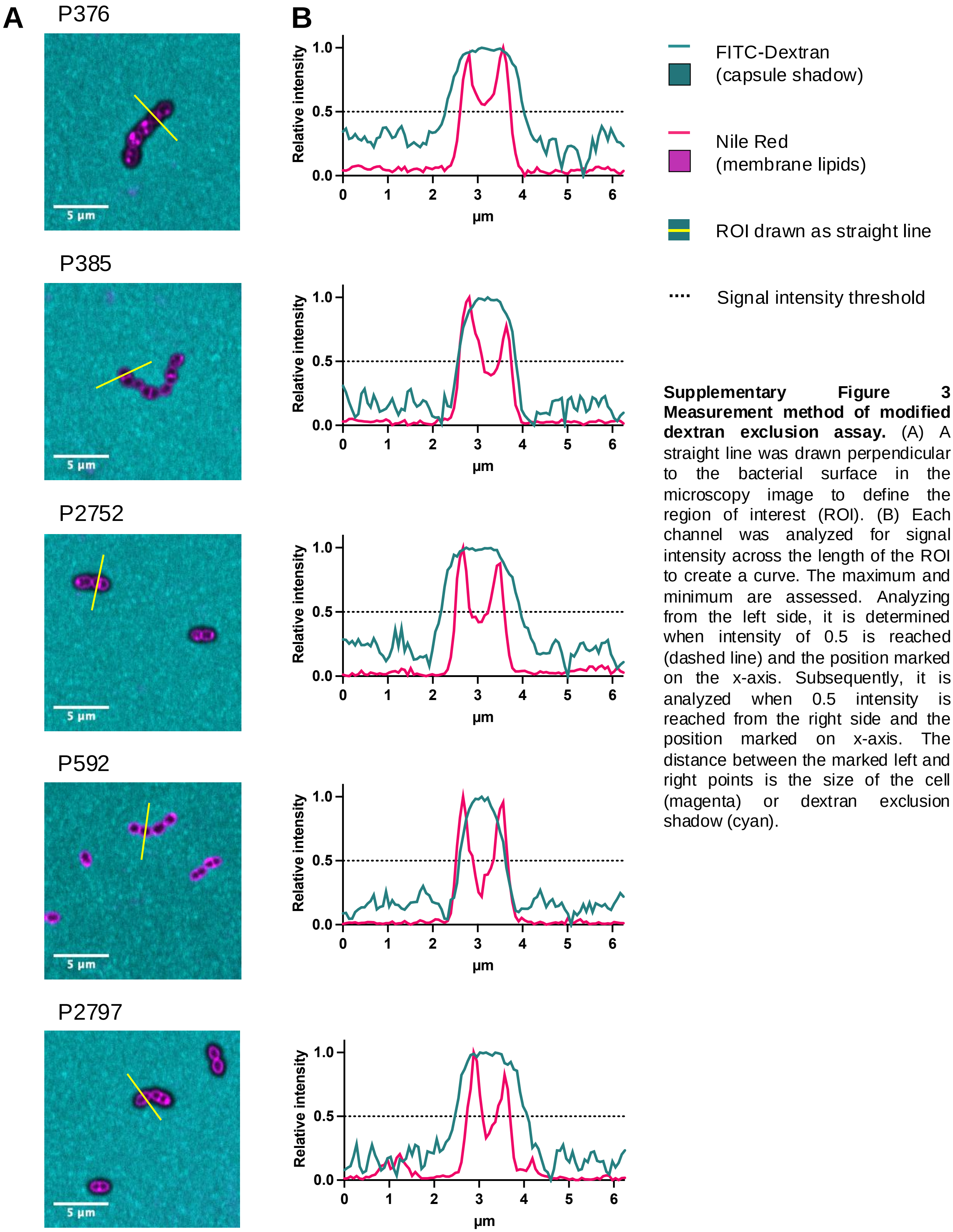

Supplementary Figure 3 Measurement method of modified dextran exclusion assay. (A) A straight line was drawn perpendicular to the bacterial surface in the microscopy image to define the region of interest (ROI). (B) Each channel was analyzed for signal intensity across the length of the ROI to create a curve. The maximum and minimum are assessed. Analyzing from the left side, it is determined when intensity of 0.5 is reached (dashed line) and the position marked on the x-axis. Subsequently, it is analyzed when 0.5 intensity is reached from the right side and the position marked on x-axis. The distance between the marked left and right points is the size of the cell (magenta) or dextran exclusion shadow (cyan).

Fig S4

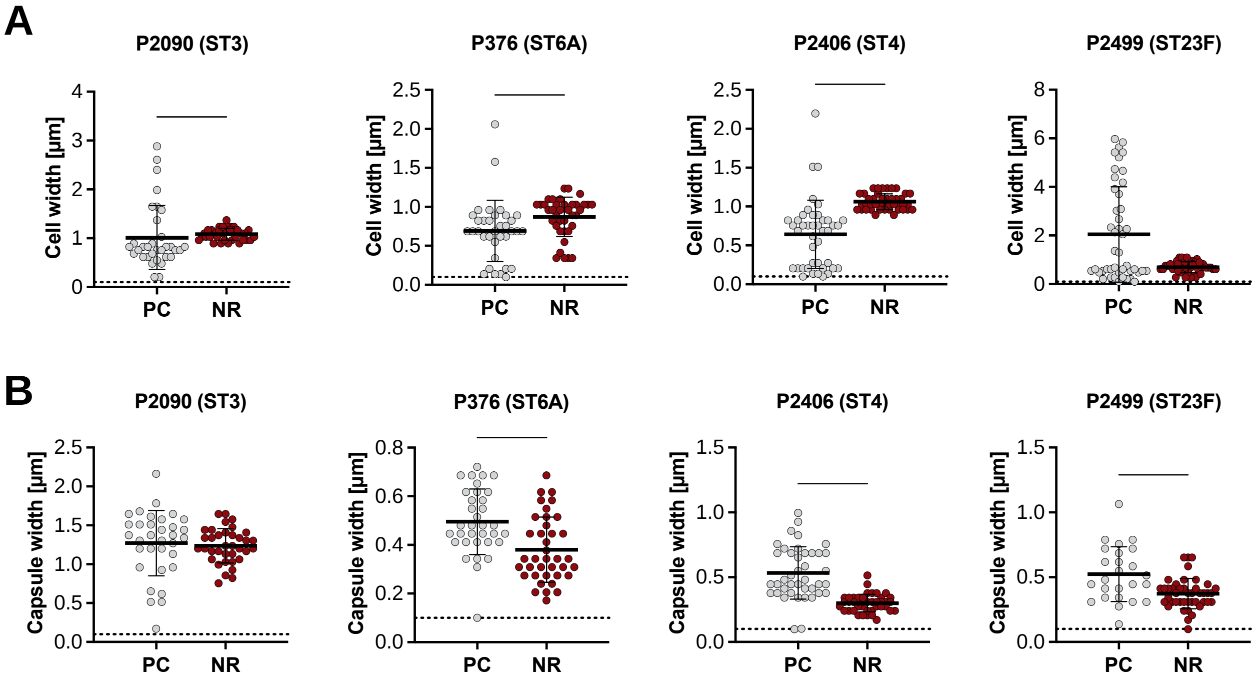

Supplementary Figure 4 Better capsule size resolution using Nile red as cell surface stain. Microscopy images were analyzed for the cell size (A) and capsule size (B). Dashed line represents limit of detection, based on the unencapsulated strain P2422. PC – Phase contrast. NR – Nile red. Data represents individual data points from one experiment. Standard deviation shown. Shapiro-Wilk test performed to test normal distribution. Unpaired, two-tailed Mann-Whitney performed. **p<0.01, ***p<0.001

Fig S5

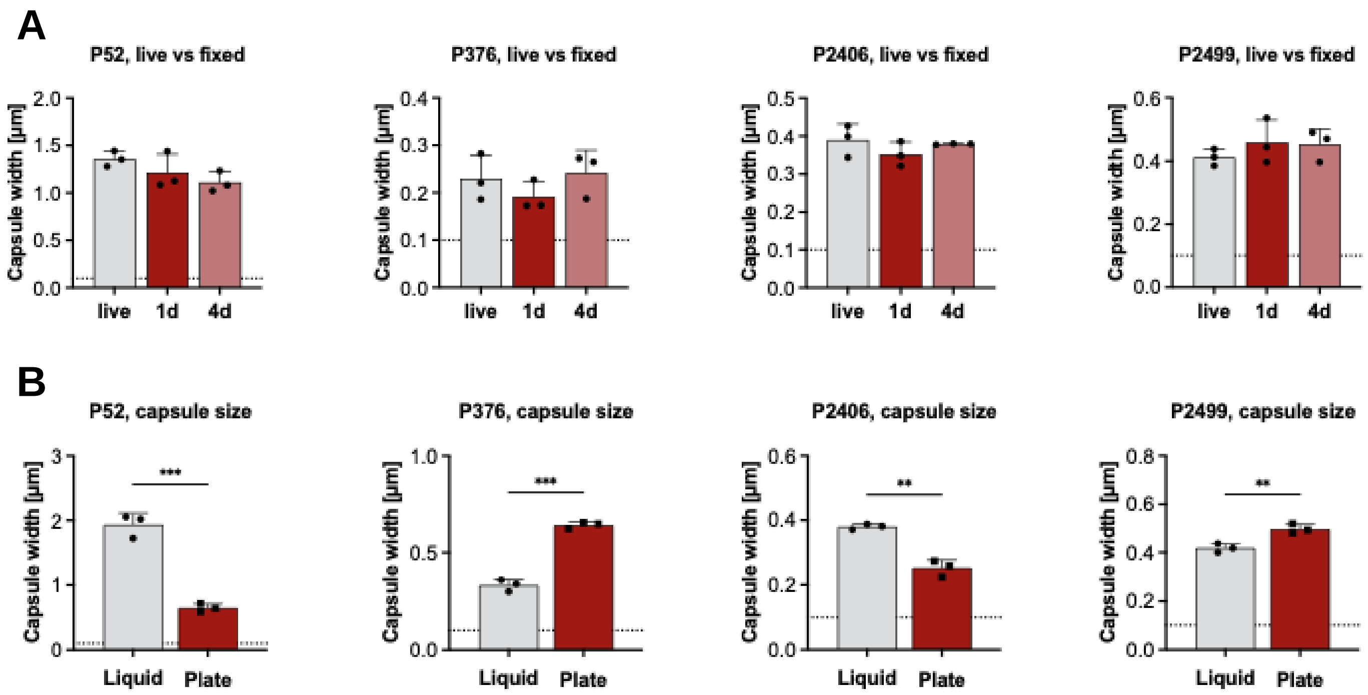

Supplementary Figure 5 Capsule size analysis at different conditions. (A) Assessment whether capsule thickness can be measured using the modified dextran exclusion assay when bacteria are fixed with lysine, glutaraldehyde and formaldehyde. The same culture was used for live and fixed bacteria. Ordinary one-way ANOVA performed compared to live cells. (B) Capsule thickness and cell size was measured using the modified dextran exclusion assay for the reference strains when grown in solid phase or liquid phase. Unpaired, two-tailed Student's t-test performed. (A & B) Dashed line represents limit of detection, based on the unencapsulated strain P2422. All data represents mean of three independent experiments with standard deviation. *p<0.05, **p<0.01, ***p<0.001

Fig S6

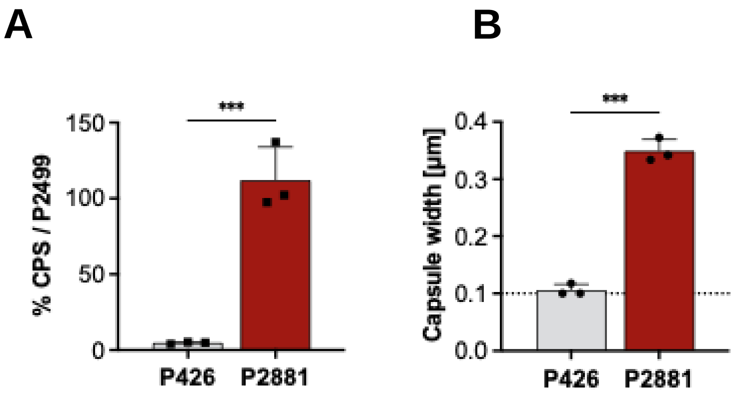

Supplementary Figure 6 CPS and capsule size analysis on serotype 23F. P426 and its descendant P2881 were analyzed for CPS analysis using immunoblot and densitometry (A) and capsule thickness using the modified dextran exclusion assay (B). Dashed line represents limit of detection, based on the unencapsulated strain P2422. (A & B) All data represents mean of three independent experiments with standard deviation. Unpaired, two-tailed Student's t-test performed. ***p<0.001

Supplementary Figure 7 Sequence alignment for CpsE of serotype 6A alignment. Page 1

```
P376    MNGKIVKSSLAIIQSFLVILLTYLLSAVRETEIVSTTAIALYILHYFVFYISDYGQDFFKRGYLIELVQTLKYILFFALA  80
P385    MNGKIVKSSLAIIQSFLVILLTYLLSAVRETEIVSTTAIALYILHYFVFYISDYGQDFFKRGYLIELVQTLKYILFFALA  80
P2752   MNGKIVKSSLAIIQSFLVILLTYLLSAVRETEIVSTTAIALYILHYFVFYISDYGQDFFKRGYLIELVQTLKYILFFALA  80
P592    MNGKIVKSSLAIIQSFLVILLTYLLSAVRETEIVSTTAIALYILHYFVFYISDYGQDFFKRGYLIELVQTLKYILFFALA  80
P2797   MNGKIVKSSLAIIQSFLVILLTYLLSAVRETEIVSTTAIALYILHYFVFYISDYGQDFFKRGYLIELVQTLKYILFFALA  80
P302    MNGKIVKSSLAIIQSFLVILLTYLLSAVREAEIVSTTAIALYILHYFVFYISDYGQDFFKRGYLIELVQTLKYILFFALA  80
P306    MNGKIVKSSLAIIQSFLVILLTYLLSAVRETEIVSTTAIALYILHYFVFYISDYGQDFFKRGYLIELVQTLKYILFFALA  80
P307    MNGKIVKSSLAIIQSFLVILLTYLLSAVREAEIVSTTAIALYILHYFVFYISDYGQDFFKRGYLIELVQTLKYILFFALA  80
P448    MNGKIVKSSLVIIQSSLVILLTYLLSTVREAKIVSTTAIALYILHYFVFYISDYGQDFFKRGYLIELVQTLKYILFFALA  80
P461    MNGKIVKSSLAIIQSFLVILLTYLLSAVREAEIVSTTAIALYILHYFVFYISDYGQDFFKRGYLIELVQTLKYILFFALA  80
P503    MNGKIVKSSLAIIQSFLVILLTYLLSAVREAEIVSTTAIALYILHYFVFYISDYGQDFFKRGYLIELVQTLKYILFFALA  80
P1859   MNGKIVKSSLVIIQSSLVILLTYLLSAVREAKIVSTTAIALYILHYFVFYISDYGQDFFKRGYLIELVQTLKYILFFALA  80

P376    ISISNFFLEDRFSISRRGMIYFLTLHALLVYVLNLFIKWYWKRAYPNFKGSKKILLLTATSRVEKVLDRLIESNEVVGEL  160
P385    ISISNFFLEDRFSISRRGMIYFLTLHALLVYVLNLFIKWYWKRAYPNFKGSKKILLLTATSRVEKVLDRLIESNEVVGEL  160
P2752   ISISNFFLEDRFSISRRGMIYFLTLHALLVYVLNLFIKWYWKRAYPNFKGSKKILLLTATSRVEKVLDRLIESNEVVGEL  160
P592    ISISNFFLEDRFSISRRGMIYFLTLHALLVYVLNLFIKWYWKRAYPNFKGSKKILLLTATSRVEKVLDRLIESNEVVGEL  160
P2797   ISISNFFLEDRFSISRRGMIYFLTLHALLVYVLNLFIKWYWKRAYPNFKGSKKILLLTATSRVEKVLDRLIESNEVVGEL  160
P302    ISISNFFLEDRFSISRRGMIYFLTLHALLVYVLNLFIKWYWKRAYPNFKGSKKILLLTATSRIEKVLDRLIESNEVVGEL  160
P306    ISISNFFLEDRFSISRRGMIYFLTLHALLVYVLNLFIKWYWKRAYPNFKGSKKILLLTATSRVEKVLDRLIESNEVVGEL  160
P307    ISISNFFLEDRFSISRRGMIYFLTLHALLVYVLNLFIKWYWKRAYPNFKGSKKILLLTATSRIEKVLDRLIESNEVVGEL  160
P448    ISISNFFLEDRFSISRRGMIYFLTLHALLVYVLNLFIKWYWKRAYPNFKGSKKILLLTATSRVEKVLDGLIESNEVVGEL  160
P461    ISISNFFLEDRFSISRRGMIYFLTLHALLVYVLNLFIKWYWKRAYPNFKGSKKILLLTATSRVEKVLDRLIESNEVVGEL  160
P503    ISISNFFLEDRFSISRRGMIYFLTLHALLVYVLNLFIKWYWKRAYPNFKGSKKILLLTATSRVEKVLDRLIESNEVVGEL  160
P1859   ISISNFFLEDRFSISRRGMIYFLTLHALLVYVLNLFIKWYWKRAYPNFKGSKKILLLTATSRVEKVLDGLIESNEVVGEL  160

P376    VAVSVLDKPDFQHDCLKVVAEGEIVNFATHEVVDEVFINLPSEKYNIGELVSQFETMGIDVTVNLNAFDRSLARNKQIRE  240
P385    VAVSVLDKPDFQHDCLKVVAEGEIVNFATHEVVDEVFINLPSEKYNIGELVSQFETMGIDVTVNLNAFDRSLARNKQIRE  240
P2752   VAVSVLDKPDFQHDCLKVVAEGEIVNFATHEVVDEVFINLPSEKYNIGELVSQFETMGIDVTVNLNAFDRSLARNKQIRE  240
P592    VAVSVLDKPDFQHDCLKVVAEGEIVNFATHEVVDEVFINLPSEKYNIGELVSQFETMGIDVTVNLNAFDRSLARNKQIRE  240
P2797   VAVSVLDKPDFQHDCLKVVAEGEIVNFATHEVVDEVFINLPSEKYNIGELVSQFETMGIDVTVNLNAFDRSLARNKQIRE  240
P302    VAVSVLDKPDFQHDYLKVVAEGEIVNFATHEVVDEVFINLPSEKYNIGELVSQFETMGIDVTVNLNAFDRSLARNKQIRE  240
P306    VAVSVLDKPDFQHDCLKVVAEGEIVNFATHEVVDEVFINLPSEKYNIGELVSQFETMGIDVTVNLNAFDRSLARNKQIRE  240
P307    VAVSVLDKPDFQHDYLKVVAEGEIVNFATHEVVDEVFINLPSEKYNIGELVSQFETMGIDVTVNLNAFDRSLARNKQIRE  240
P448    VAVSVLDKPDFQHDYLKIVAEGEIVNFATHEVVDEVFINLPSEKYNIGELVSQFETMGIDVTVNLNAFDRSLARNKQIRK  240
P461    VAVSVLDKPDFQHDCLKVVAEGEIVNFATHEVVDEVFINLPSEKYNIGELVSQFETMGIDVTVNLNAFDRSLARNKQIRE  240
P503    VAVSVLDKPDFQHDCLKVVAEGEIVNFATHEVVDEVFINLPSEKYNIGELVSQFETMGIDVTVNLNAFDRSLARNKQIRE  240
P1859   VAVSVLDKPDFQHDYLKIVAEGEIVNFATHEVVDEVFINLPSEKYNIGELVSQFETMGIDVTVNLNAFDRSLARNKQIRK  240
```

Supplementary Figure 7 Sequence alignment for CpsE of serotype 6A alignment. Page 2

P376	MAGLNVVTFSTTFYKTSHVIAKRIIDIVGALVGLILCGLVSIVLVPLIRKDGGSAIFAQTRIGKNGRQFTFYKFRSMCVD	320
P385	MAGLNVVTFSTTFYKTSHVIAKRIIDIVGALVGLILCGLVSIVLVPLIRKDGGSAIIAQTRIGKNGRQFTFYKFRSMCVD	320
P2752	MAGLNVVTFSTTFYKTSHVIAKRIIDIVGALVGLILCGLVSIVLVPLIRKDGGSAIFAQTRIGKNGRQFTFYKFRSMCVD	320
P592	MAGLNVVTFSTTFYKTSHVIAKRIIDIVGALVGLILCGLVSIVLVPLIRKDGGSAIFAQTRIGKNGRQFTFYKFRSMCVD	320
P2797	MAGLNVVTFSTTFYKTSHVIAKRIIDIVGALVGLILCGLVSIVLVPLIRKDGGSAIFAQTRIGKNGRQFTFYKFRSMCVD	320
P302	MAGLNVVTFSTTFYKTSHVIAKRIIDIVGALVGLILCGLVSIVLVPLIRKDGGSAIFAQTRIGKNGRQFTFYKFRSMCVD	320
P306	MAGLNVVTFSTTFYKTSHVIAKRIIDIVGALVGLILCGLVSIVLVPLIRKDGGSAIFAQTRIGKNGRQFTFYKFRSMCVD	320
P307	MAGLNVVTFSTTFYKTSHVIAKRIIDIVGALVGLILCGLVSIVLVPLIRKDGGSAIFAQTRIGKNGRQFTFYKFRSMCVD	320
P448	MAGLNVVTFSTTFYKTSHVIAKRIIDIVGALVGLILCGLVSIVLVPLIRKDGGSAIFAQTRIGKNGRQFTFYKFRSMCVD	320
P461	MAGLNVVTFSTTFYKTSHVIAKRIIDIVGALVGLILCGLVSIVLVPLIRKDGGSAIFAQTRIGKNGRQFTFYKFRSMCVD	320
P503	MAGLNVVTFSTTFYKTSHVIAKRIIDIVGALVGLILCGLVSIVLVPLIRKDGGSAIFAQTRIGKNGRQFTFYKFRSMCVD	320
P1859	MAGLNVVTFSTTFYKTSHVIAKRIIDIVGALVGLILCGLVSIVLVPLIRKDGGSAIFAQTRIGKNGRQFTFYKFRSMCVD	320

P376	AEAKKRELMEQNTMQGGMFKVDDDPRITKIGRFIRKTSLDELPQFYNVLKGDMSLVGTRPPTVDEYEHYTPEQKRRLSFK	400
P385	AEAKKRELMEQNTMQGGMFKVDDDPRITKIGRFIRKTSLDELPQFYNVLKGDMSLVGTRPPTVDEYEHYTPEQKRRLSFK	400
P2752	AEAKKRELMEQNTMQGGMFKVDDDPRITKIGRFIRKTSLDELPQFYNVLKGDMSLVGTRPPTVDEYEHYTPEQKRRLSFK	400
P592	TEAKKRELMEQNTMQGGMFKVDDDPRITKIGRFIRKTSLDELPQFYNVLKGDMSLVGTRPPTVDEYEHYTPEQKRRLSFK	400
P2797	AEAKKRELMEQNTMQGGMFKVDDDPRITKIGRFIRKTSLDELPQFYNVLKGDMSLVGTRPPTVDEYEHYTPEQKRRLSFK	400
P302	AEAKKRELMEQNTMQGGMFKVDDDPRITKIGRFIRKTSLDELPQFYNVLKGDMSLVGTRPPTVDEYEHYTPEQKRRLSFK	400
P306	AEAKKRELMEQNTMQGGMFKVDDDPRITKIGRFIRKTSLDELPQFYNVLKGDMSLVGTRPPTVDEYEHYTPEQKRRLSFK	400
P307	AEAKKRELMEQNTMQGGMFKVDDDPRITKIGRFIRKTSLDELPQFYNVLKGDMSLVGTRPPTVDEYEHYTPEQKRRLSFK	400
P448	AEAKKRELMEQNTMQGGMFKVDDDPRITKIGCFIRKTSLDELPQFYNVLKGDMSLVGTRPPTVDEYEHYTPEQKRRLSFK	400
P461	AEAKKRELMEQNTMQGGMFKVDDDPRITKIGRFIRKTSLDELPQFYNVLKGDMSLVGTRPPTVDEYEHYTPEQKRRLSFK	400
P503	AEAKKRELMEQNTMQGGMFKVDDDPRITKIGRFIRKTSLDELPQFYNVLKGDMSLVGTRPPTVDEYEHYTPEQKRRLSFK	400
P1859	AEAKKRELMEQNTMQGGMFKVDDDPRITKIGCFIRKTSLDELPQFYNVLKGDMSLVGTRPPTVDEYEHYTPEQKRRLSFK	400

P376	PGITGLWQVSGRSEIKNFDEVVKLDVAYINGWTIWKDIEILLKTVKVVFMRDGAK	455
P385	PGITGLWQVSGRSEIKNFDEVVKLDVAYINGWTIWKDIEILLKTVKVVFMRDGAK	455
P2752	PGITGLWQVSGRSEIKNFDEVVKLDVAYINGWTIWKDIEILLKTVKVVFMRDGAK	455
P592	PGITGLWQVSGRSEIKNFDEVVKLDVAYINGWTIWKDIEILLKTVKVVFMRDGAK	455
P2797	PGITGLWQVSGRSEIKNFDEVVKLDVAYINGWTIWKDIEILLKTVKVVFMRDGAK	455
P302	PGITGLWQVSGRSEIKNFDEVVKLDVAYINGWTIWKDIEILLKTVKVVFMRDGAK	455
P306	PGITGLWQVSGRSEIKNFDEVVKLDVAYINGWTIWKDIEILLKTVKVVFMRDGAK	455
P307	PGITGLWQVSGRSEIKNFDEVVKLDVAYINGWTIWKDIEILLKTVKVVFMRDGAK	455
P448	PGITGLWQVSGRSEIKNFDEVVKLDVAYIDDWTIWKDIEILLKTVKVVLMKDGAK	455
P461	PGITGLWQVSGRSEIKNFDEVVKLDVAYINGWTIWKDIEILLKTVKVVFMRDGAK	455
P503	PGITGLWQVSGRSEIKNFDEVVKLDVAYINGWTIWKDIEILLKTVKVVFMRDGAK	455
P1859	PGITGLWQVSGRSEIKNFDEVVKLDVAYIDDWTIWKDIEILLKTVKVVLMKDGAK	455

Supplementary Figure 8 Sequence alignment for CpsE of serotype 23F alignment. Page 1

P2499	MNGKILRSSLAIIQSFLVILLTYLLSAVRETEIVSTTAIALYILHYFVFYISDYGQDFFKRRYLIELVQTLKYILFFALA	80
P426	MNGKILRSSLAIIQSFLVILLTYLLSAVRETEIVSTTAIALYILHYFVFYISDYGQDFFKRRYLIELVQTLKYILFFALA	80
P2881	MNGKILRSSLAIIQSFLVILLTYLLSAVRETEIVSTTAIALYILHYFVFYISDYGQDFFKRRYLIELVQTLKYILFFALA	80
P5	MNEKILRSSLAIIQSFLVILLTYLLSAVRETEIVSTTAIALCILHYFVFYISDYGQDFFKRRYLIELVQTLKYILFFALA	80
P19	MNGKILRSSLAIIQSFLVILLTYLLSAVRETEIVSTTAIALYILHYFVFYISDYGQDFFKRRYLIELVQTLKYILFFALA	80
P438	MNEKILRSSLAIIQSFLVILLTYLLSAVRETEIVSTTAIALCILHYFVFYISDYGQDFFKRRYLIELVQTLKYILFFALA	80
P822	MNEKILRSSLAIIQSFLVILLTYLLSAVRETEIVSTTAIALYILHYFVFYISDYGQDFFKRRYLIELVQTLKYILFFALA	80
P1091	MNGKILRSSLAIIQSFLVILLTYLLSAVRETEIVSTTAIALYILHYFVFYISDYGQDFFKRRYLIELVQTLKYILFFALA	80
P1237	MNGKILRSSLAIIQSFLVILLTYLLSAVRETEIVSTTAIALYILHYFVFYISDYGQDFFKRRYLIELVQTLKYILFFALA	80
P1460	MNGKILRSSLAIIQSFLVILLTYLLSAVRETEIVSTTAIALYILHYFVFYISDYGQDFFKRRYLIELVQTLKYILFFALA	80
P1863	MNGKILRSSLAIIQSFLVILLTYLLSAVRETEIVSTTAIALYILHYFVFYISDYGQDFFKRRYLIELVQTLKYILFFALA	80
P1900	MNGKIVRSSLAIIQSFLVILLTYLLSAVRETEIVSTTAIALYILHYFVFYISDYGQDFFKRRYLIELVQTLKYILFFALA	80

P2499	IGISNFFLEDRFSISRRGMIYFLTLHALLVYVLNLFIKWYWKRAYPNFKGSKKILLLTATSRVEKVLDRLIESNEVVGKL	160
P426	IGISNFFLEDRFSISRRGMIYFLTLHALLVYVLNLFIKWYWKRAYPNFKGSKKILLLTATSRVEKVLDRLIESNEVVGKL	160
P2881	IGISNFFLEDRFSISRRGMIYFLTLHALLVYVLNLFIKWYWKRAYPNFKGSKKILLLTATSRVEKVLDRLIESNEVVGKL	160
P5	IGISNFFLEDRFSISRRGMIYFLTLHALLVYVLNLFIKWYWKRAYPNFKGSKKILLLTATSRVEKVLDRLIESNEVVGKL	160
P19	IGISNFFLEDRFSISRRGMIYFLTLHALLVYVLNLFIKWYWKRAYPNFKGSKKILLLTATSRVEKVLDRLIESNEVVGKL	160
P438	IGISNFFLEDRFSISRRGMIYFLTLHALLVYVLNLFIKWYWKRAYPNFKGSKKILLLTATSRVEKVLDRLIESNEVVGKL	160
P822	IGISNFFLEDRFSISRRGMIYFLTLHALLVYVLNLFIKWYWKRAYPNFKGSKKILLLTATSRVEKVLDRLIESNEVVGKL	160
P1091	IGISNFFLEDRFSISRRGMIYFLTLHALLVYVLNLFIKWYWKRAYPNFKGSKKILLLTATSRVEKVLDRLIESNEVVGKL	160
P1237	IGISNFFLEDRFSISRRGMIYFLTLHALLVYVLNLFIKWYWKRAYPNFKGSKKILLLTATSRVEKVLDRLIESNEVVGKL	160
P1460	IGISNFFLEDRFSISRRGMIYFLTLHALLVYVLNLFIKWYWKRAYPNFKGSKKILLLTATSRVEKVLDRLIESNEVVGKL	160
P1863	IGISNFFLEDRFSISRRGMIYFLTLHALLVYVLNLFIKWYWKRAYPNFKGSKKILLLTATSRVEKVLDRLIESNEVVGKL	160
P1900	ISISNFFLEDRFSISRRGMIYFLTLHALLVYVLNLFIKWYWKRAYPNFKGSKKILLLTATSRVEKVLDRLIESNEVVGKL	160

P2499	VAVSVLDKPDFQHDCLKVVAEGEIVNFATHEVVDEVFINLPSEKYNIGELVSQFETMGIDVIVNLNAFDRSLARNKQIRE	240
P426	VAVSVLDKPDFQHDCLKVVAEGEIVNFATHEVVDEVFINLLSEKYNIGELVSQFETMGIDVIVNLNAFDRSLARNKQIRE	240
P2881	VAVSVLDKPDFQHDCLKVVAEGEIVNFATHEVVDEVFINLPSEKYNIGELVSQFETMGIDVIVNLNAFDRSLARNKQIRE	240
P5	VAVSVLDKPDFQHDCLKVVAEGEIVNFATHEVVDEVFINLPSEKYNIGELVSQFETMGIDVIVNLNAFDRSLARNKQIRE	240
P19	VAVSVLDKPDFQHDCLKVVAEGEIVNFATHEVVDEVFINLPSEKYNIGELVSQFETMGIDVIVNLNAFDRSLARNKQIRE	240
P438	VAVSVLDKPDFQHDCLKVVAEGEIVNFATHEVVDEVFINLPSEKYNIGELVSQFETMGIDVIVNLNAFDRSLARNKQIRE	240
P822	VAVSVLDKPDFQHDCLKVVAEGEIVNFATHEVVDEVFINLPGEKYNIGELVSQFETMGIDVIVNLNAFDRSLARNKQIRE	240
P1091	VAVSVLDKPDFQHDCLKVVAEGEIVNFATHEVVDEVFINLPSEKYNIGELVSQFETMGIDVIVNLNAFDRSLARNKQIRE	240
P1237	VAVSVLDKPDFQHDCLKVVAEGEIVNFATHEVVDEVFINLPSEKYNIGELVSQFETMGIDVIVNLNAFDRSLARNKQIRE	240
P1460	VAVSVLDKPDFQHDCLKVVAEGEIVNFATHEVVDEVFINLPSEKYNIGELVSQFETMGIDVIVNLNAFDRSLARNKQIRE	240
P1863	VAVSVLDKPDFQHDCLKVVAEGEIVNFATHEVVDEVFINLPSEKYNIGELVSQFETMGIDVIVNLNAFDRSLARNKQIRE	240
P1900	VAVSVLDKPDFQHDCLKVVAEGEIVNFATHEVVDEVFINLPSEKYNIGELVSQFETMGIDVIVNLNAFDRSLARNKQIRE	240

Supplementary Figure 8 Sequence alignment for CpsE of serotype 23F alignment. Page 2

```
P2499   MAGLNVVTFSTTFYKTSHVIAKRIIDIVGALVGLILCGLVSIVLVPLIRKDGGSAIFAQTRIGKNGRQFTFYKFRSMCVD 320
P426    MAGLNVVTFSTTFYKTSHVIAKRIIDIVGALVGLILCGLVSIVLVPLIRKDGGSAIFAQTRIGKNGRQFTFYKFRSMCVD 320
P2881   MAGLNVVTFSTTFYKTSHVIAKRIIDIVGALVGLILCGLVSIVLVPLIRKDGGSAIFAQTRIGKNGRQFTFYKFRSMCVD 320
P5      MAGLNVVTFSTTFYKTSHVIAKRIIDIVGALVGLILCGLVSIVLVPLIRKDGGSAIFAQTRIGKNGRQFTFYKFRSMCVD 320
P19     MAGLNVVTFSTTFYKTSHVIAKRIIDIVGALVGLILCGLVSIVLVPLIRKDGGSAIFAQTRIGKNGRQFTFYKFRSMCVD 320
P438    MAGLNVVTFSTTFYKTSHVIAKRIIDIVGALVGLILCGLVSIVLVPLIRKDGGSAIFAQTRIGKNGRQFTFYKFRSMCVD 320
P822    MAGLNVVTFSTTFYKTSHVIAKRIIDIVGALVGLILCGLVSIVLVPLIRKDGGSAIFAQTRIGKNGRQFTFYKFRSMCVD 320
P1091   MAGLNVVTFSTTFYKTSHVIAKRIIDIVGALVGLILCGLVSIVLVPLIRKDGGSAIFAQTRIGKNGRQFTFYKFRSMCVD 320
P1237   MAGLNVVTFSTTFYKTSHVIAKRIIDIVGALVGLILCGLVSIVLVPLIRKDGGSAIFAQTRIGKNGRQFTFYKFRSMCVD 320
P1460   MAGLNVVTFSTTFYKTSHVIAKRIIDIVGALVGLILCGLVSIVLVPLIRKDGGSAIFAQTRIGKNGRQFTFYKFRSMCVD 320
P1863   MAGLNVVTFSTTFYKTSHVIAKRIIDIVGALVGLILCGLVSIVLVPLIRKDGGSAIFAQTRIGKNGRQFTFYKFRSMCVD 320
P1900   MAGLNVVTFSTTFYKTSHVIAKRIIDIVGALVGLILCGLVSIVLVPLIRKDGGSAIFAQTRIGKNGRQFTFYKFRSMCVD 320

P2499   AEAKKRELMEQNTMQGGMFKVDDDPRITKIGCFIRKTSLDELPQFYNVLKGDMSLVGTRPPTVDEYEHYTPEQKRRLSFK 400
P426    AEAKKRELMEQNTMQGGMFKVDDDPRITKIGCFIRKTSLDELPQFYNVLKGDMSLVGTRPPTVDEYEHYTPEQKRRLSFK 400
P2881   AEAKKRELMEQNTMQGGMFKVDDDPRITKIGCFIRKTSLDELPQFYNVLKGDMSLVGTRPPTVDEYEHYTPEQKRRLSFK 400
P5      AEAKKRELMEQNTMQGGMFKVDDDPRITKIGCFIRKTSLDELPQFYNVLKGDMSLVGTRPPTVDEYEHYTPEQKRRLSFK 400
P19     AEAKKRELMEQNTMQGGMFKVDDDPRITKIGCFIRKTSLDELPQFYNVLKGDMSLVGTRPPTVDEYEHYTPEQKRRLSFK 400
P438    AEAKKRELMEQNTMQGGMFKVDDDPRITKIGCFIRKTSLDELPQFYNVLKGDMSLVGTRPPTVDEYEHYTPEQKRRLSFK 400
P822    AEAKKRELMEQNTMQGGMFKVDDDPRITKIGCFIRKTSLDELPQFYNVLKGDMSLVGTRPPTVDEYEHYTPEQKRRLSFK 400
P1091   AEAKKRELMEQNTMQGGMFKVDDDPRITKIGCFIRKTSLDELPQFYNVLKGDMSLVGTRPPTVDEYEHYTPEQKRRLSFK 400
P1237   AEAKKRELMEQNTMQGGMFKVDDDPRITKIGCFIRKTSLDELPQFYNVLKGDMSLVGTRPPTVDEYEHYTPEQKRRLSFK 400
P1460   AEAKKRELMEQNTMQGGMFKVDDDPRITKIGCFIRKTSLDELPQFYNVLKGDMSLVGTRPPTVDEYEHYTPEQKRRLSFK 400
P1863   AEAKKRELMEQNTMQGGMFKVDDDPRITKIGCFIRKTSLDELPQFYNVLKGDMSLVGTRPPTVDEYEHYTPEQKRRLSFK 400
P1900   AEAKKRELMEQNTMQGGMFKVDDDPRITKIGCFIRKTSLDELPQFYNVLKGDMSLVGTRPPTVDEYEHYTPEQKRRLSFK 400

P2499   PGITGLWQVSGRSEIKNFDEVVKLDVAYIDGWTIWKDIEILLKTVKVVFMRDGAK 455
P426    PGITGLWQVSGRSEIKNFDEVVKLDVAYIDGWTIWKDIEILLKTVKVVFMRDGAK 455
P2881   PGITGLWQVSGRSEIKNFDEVVKLDVAYIDGWTIWKDIEILLKTVKVVFMRDGAK 455
P5      PGITGLWQVSGRSEIKNFDEVVKLDVAYIDGWTIWKDIEILLKTVKVVFMRDGAK 455
P19     PGITGLWQVSGRSEIKNFDEVVKLDVAYIDGWTIWKDIEILLKTVKVVFMRDGAK 455
P438    PGITGLWQVSGRSEIKNFDEVVKLDVAYIDGWTIWKDIEILLKTVKVVFMRDGAK 455
P822    PGITGLWQVSGRSEIKNFDEVVKLDVAYIDGWTIWKDIEILLKTVKVVFMRDGAK 455
P1091   PGITGLWQVSGRSEIKNFDEVVKLDVAYIDGWTIWKDIEILLKTVKVVFMRDGAK 455
P1237   PGITGLWQVSGRSEIKNFDEVVKLDVAYIDGWTIWKDIEILLKTVKVVFMRDGAK 455
P1460   PGITGLWQVSGRSEIKNFDEVVKLDVAYIDGWTIWKDIEILLKTVKVVFMRDGAK 455
P1863   PGITGLWQVSGRSEIKNFDEVVKLDVAYIDGWTIWKDIEILLKTVKVVFMRDGAK 455
P1900   PGITGLWQVSGRSEIKNFDEVVKLDVAYIDGWTIWKDIEILLKTVKVVFMRDGAK 455
```

Fig S9

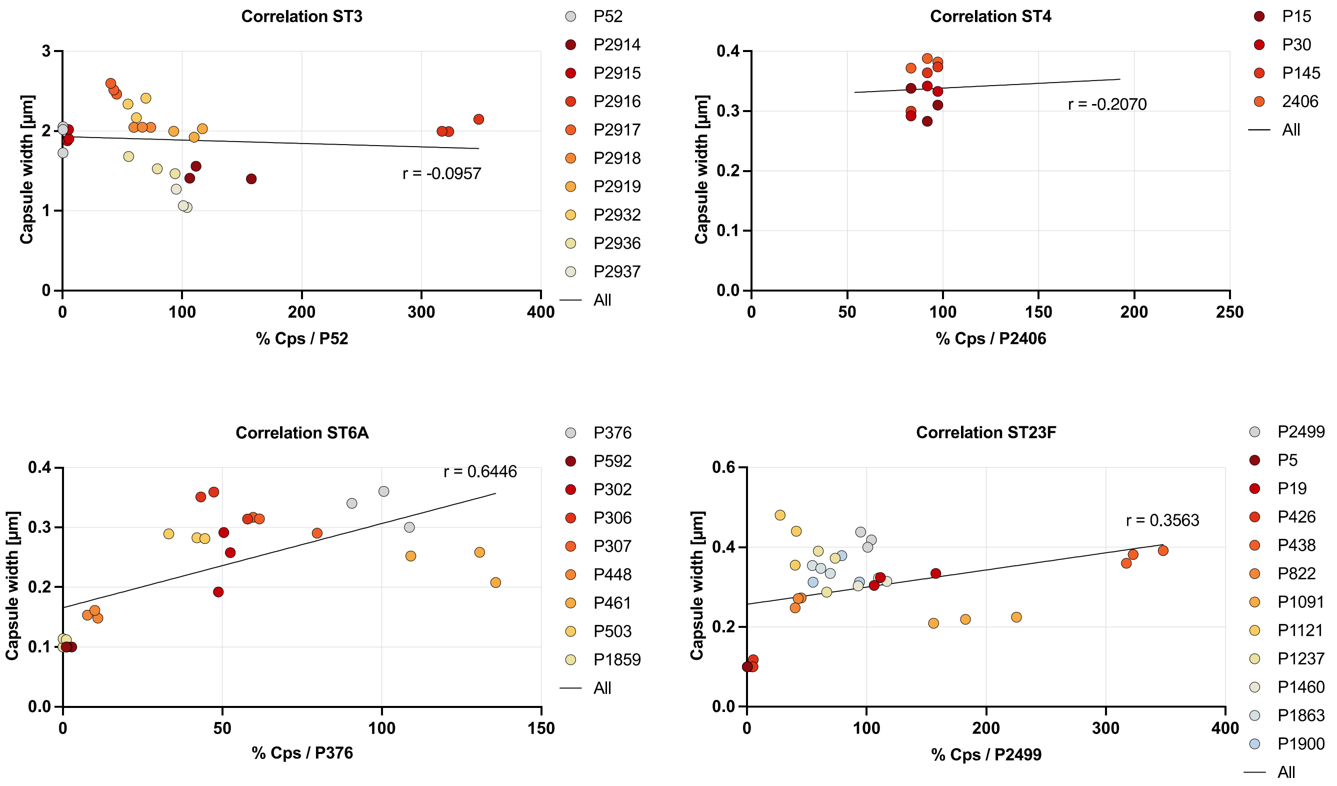

Supplementary Figure 9 Correlation analysis between CPS and Capsule width. Correlation analysis was performed for the relationship between the amount of CPS produced and capsule thickness. Independent experiments of each strain are plotted, with linear regression shown. Correlation value r = 1 shows maximum positive correlation, 0 no correlation, -1 maximum negative correlation.
